# The role of endoscopic versus microsurgical techniques in optic canal decompression: a meta-analysis of randomized controlled trials

**DOI:** 10.3389/fneur.2025.1691086

**Published:** 2025-11-25

**Authors:** Feng Lin, Peng Song

**Affiliations:** 1 Department of Neurosurgery, Guangzhou Hospital of Integrated Traditional and Western Medicine, Guangzhou, China; 2 Department of Neurosurgery, Guangyuan Central Hospital, Sichuan, China

**Keywords:** compressive optic neuropathy, endoscopic decompression, microsurgical decompression, optic canal surgery, visual acuity improvement

## Abstract

**Background:**

The ideal surgical technique for optic canal decompression (OCD) in cases of compressive optic neuropathy continues to be the subject of contention. Endoscopic and microsurgical OCD procedures have demonstrated encouraging outcomes; however, their comparative efficacy in enhancing visual acuity and post-op complications remains unclear. This meta-analysis thus evaluated the safety and efficacy of these methods across different circumstances.

**Methods:**

A systematic review and meta-analysis were performed in accordance with PRISMA guidelines. Relevant randomized controlled trials (RCTs) were identified through PubMed, Embase, Cochrane Library, Scopus, and Web of Science. Studies assessed endoscopic and microsurgical decompression techniques for compressive ocular neuropathies. Statistical analyses were conducted using RevMan 5.4. A fixed-effects model was applied due to minimal heterogeneity (*I*^2^ = 0%), and statistical significance was defined as a *p*-value of <0.05.

**Results:**

A total of seven studies (*n* = 194 participants) were incorporated. Endoscopic techniques demonstrated considerable enhancements in visual acuity, especially for medial canal disorders (RR = 2.01; *p* < 0.00001). Microsurgical techniques gave superior circumferential decompression, up to 252.8° with pterional craniotomy, in contrast to 124.6° attained with endoscopic methods. Both procedures demonstrated minimal complication rates, with no substantial variations in postoperative cerebrospinal fluid (CSF) leakage or necessity for reoperation. Funnel plots suggested negligible publication bias, and sensitivity analysis validated the strength of findings.

**Conclusion:**

Both endoscopic and microsurgical techniques were effective for OCD, with endoscopic methods providing the least invasive advantages and microsurgical approaches excelling in complex diseases necessitating considerable decompression.

**Systematic review registration:**

https://www.crd.york.ac.uk/prospero/, identifier CRD420251078576.

## Introduction

Optic canal decompression is a surgical procedure aimed at relieving pressure on the optic nerve that may be caused by trauma, tumors, inflammatory diseases, or congenital defects (1). The optic nerve and ophthalmic artery traverse this optic canal, a narrow bony passage formed by the two struts of the sphenoid bone (2, 3). This canal is divided into intraorbital, intracanalicular, and intracranial segments, each of which may be affected by different injury mechanisms. Among these, the intracanalicular segment is the most constrained, rendering it particularly vulnerable to compression from trauma, space-occupying lesions, and vascular compromise, requiring targeted decompression procedures (4–6).

Over the years, multiple surgical approaches have been utilized for OCD, including transorbital, transnasal, transethmoid, transantral, and craniotomy techniques (7). Traditionally, microsurgical approaches, such as pterional, supraorbital, and frontotemporal routes, have been the standard for OCD, providing direct visualization and precise manipulation of the optic nerve. These techniques, however, are associated with higher surgical morbidity, brain retraction injury, CSF leakage, and prolonged recovery times (8).

Advancements in minimally invasive endoscopic techniques, particularly the endoscopic endonasal approach (EEA), have introduced a viable alternative to traditional transcranial procedures (9). EOCD provides direct access to the optic canal without needing extensive cranial exposure, reducing the risk of CSF leaks, surgical trauma, and prolonged hospitalization. It is particularly effective for medially located lesions such as sphenoidal tumors or traumatic optic neuropathy. Despite its advantages, EOCD has limitations, including restricted access to the lateral optic canal, challenges in achieving hemostasis, and a steep learning curve (10, 11).

Microsurgical OCD, on the other hand, remains a preferred choice in cases requiring extensive exposure, tumor resection, or complex neurovascular intervention. It is particularly advantageous in challenging anatomical scenarios such as intracranial tumors, vascular malformations, and severe traumatic injuries (7). However, its higher risk of CSF leaks, brain edema, and longer recovery periods necessitate careful patient selection.

Despite the growing adoption of endoscopic approaches, the superiority of one technique over the other remains a subject of debate. Multiple RCTs have compared microsurgical and endoscopic OCD, but their findings have been inconclusive due to variations in surgical expertise, patient pathology, and anatomical considerations. This meta-analysis evaluated the comparative safety and efficacy of endoscopic and microsurgical optic canal decompression in patients with compressive optic neuropathy.

## Materials and methods

### Study design and search strategy

A comprehensive literature search was conducted across five electronic databases: PubMed, Embase, Cochrane Library, Scopus, and Web of Science from database inception to 30 June 2025. The search strategy incorporated both Medical Subject Headings (MeSH) and free-text keywords, using Boolean operators to maximize sensitivity and specificity.

The core PubMed search string was as follows and was adapted appropriately for the other databases: (“optic canal decompression”[MeSH] OR “optic nerve decompression” OR “optic canal surgery” OR “optic canal opening” OR “optic canal decompression procedure”) AND (“endoscopic” OR “endonasal” OR “transnasal” OR “transethmoidal”) AND (“microsurgical” OR “transcranial” OR “pterional” OR “craniotomy”) AND (“randomized controlled trial” OR “randomized” OR “RCT” OR “clinical trial”) AND (“meta-analysis” OR “systematic review” OR “comparative study”).

Additional synonyms and spelling variants (e.g., optic nerve decompression, optic canal opening, EEA, and transcranial optic canal decompression) were included to ensure broad coverage. No language or date restrictions were applied during the initial search.

All retrieved records were reviewed, duplicate records were removed, and two independent reviewers screened titles and abstracts according to pre-specified inclusion and exclusion criteria. Full texts of potentially eligible studies were retrieved and assessed for eligibility. Reference lists of the included studies and relevant reviews were also manually screened to identify additional eligible articles.

**Figure 1:**
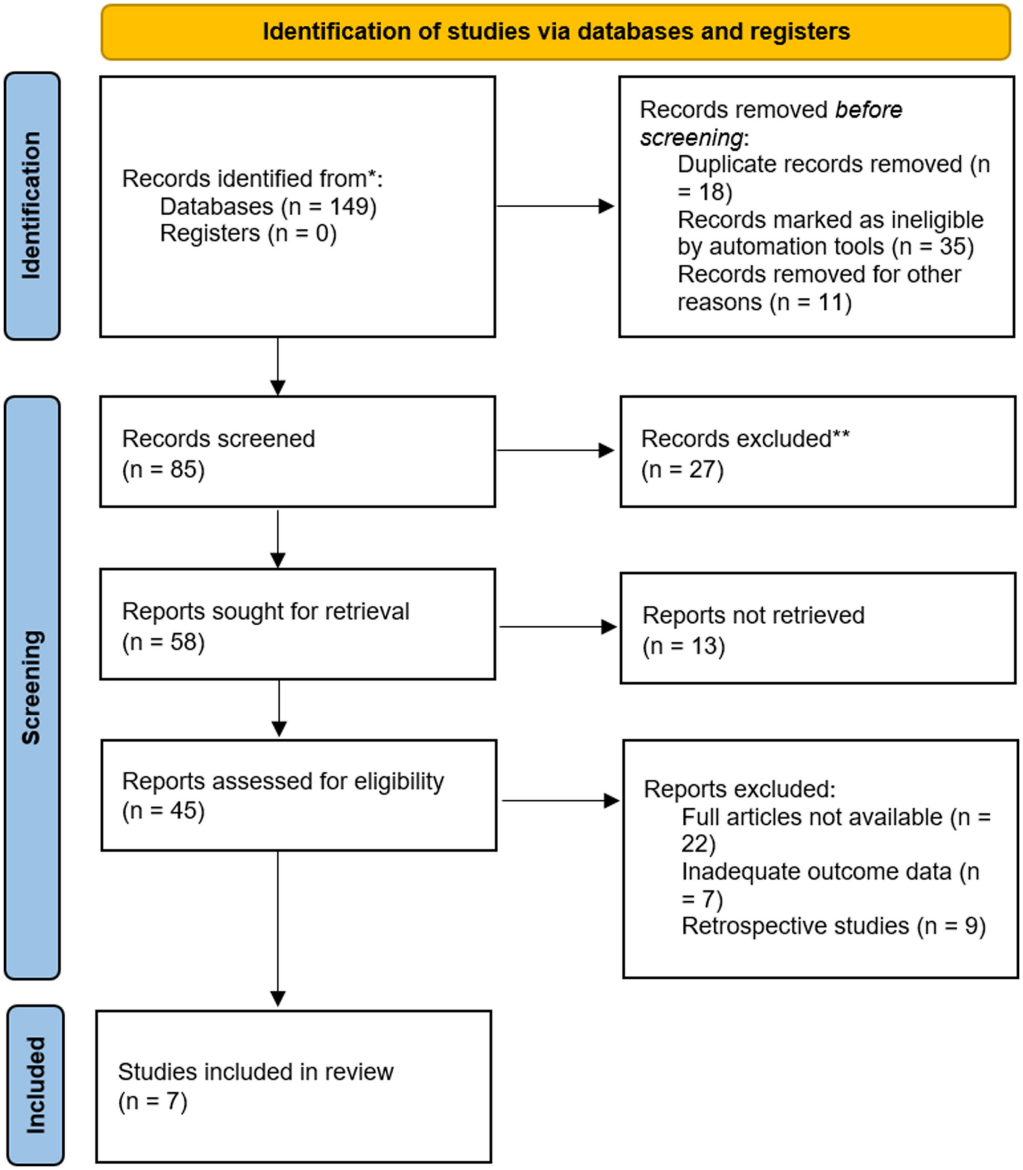
PRISMA flow diagram.

In total, 149 records were identified. After duplicate removal and eligibility assessment, seven RCTs met the inclusion criteria and were included in the final quantitative synthesis (Figure 1).

### PICO statement

This study aims to systematically evaluate and compare the safety, efficacy, and anatomical decompression of endoscopic versus microsurgical optic canal decompression in patients with compressive optic neuropathy. Specifically, the PICO framework was as follows: Population (P): patients with compressive optic neuropathy (traumatic or tumor-related); Intervention (I): endoscopic optic canal decompression; Comparison (C): microsurgical optic canal decompression; and Outcome (O): visual acuity improvement, complication rates, and circumferential decompression.

### Eligibility criteria

Studies were included if they met the following eligibility requirements:

- Design of studies: RCTs.
- Population: Patients suffering trauma, tumors, or compressive optic neuropathy leading to OCD.
- Endoscopic optic canal decompression (EEA-based procedures) against MOCD (transcranial approaches).
- Studies reporting at least one main or secondary result (exclusively detailed below).
- Language: Written in English.

### Exclusion criteria

Studies were excluded based on the following criteria:

- Studies without randomizing (case series, cohort studies, and retroactive analysis).
- Studies involving animals or cadaveric studies.
- Research without pertinent clinical result data.

### Outcome measures

The primary and secondary outcome measures were predefined before analysis.

1) Primary outcome: Postoperative improvement in visual acuity.
2) Secondary outcomes: Circumferential decompression angle, cerebrospinal fluid leakage rate, and reoperation rate.

These outcomes were selected based on their clinical relevance and frequency of reporting across the included randomized controlled trials. Effect estimates were calculated using risk ratios (RRs) and 95% confidence intervals (CIs) for dichotomous variables, and mean differences for continuous variables.

### Data extraction and quality assessment

Two separate reviewers gathered the following information using the same data extraction form:

- Characteristics of studies: Author(s), publication year, study design, and sample size.
- Characteristics of patients: Age, gender, preoperative sight, and surgical indication.
- Surgical methods: Type of microsurgical or endoscopic technique applied.
- Principal results: Visual acuity improvement and recovery of optic nerve function.
- Secondary effects included hospital length of stay, incidence of CSF, intraoperative and postoperative problems, and necessity for reoperation.
- The quality of the included RCTs was evaluated using the Cochrane Risk of Bias 2.0 (RoB 2.0) instrument, assessing biases pertaining to randomization, blinding, attrition, and reporting. Standardized criteria let studies be classified as low risk, high risk, or unknown risk of bias.

### Statistical analysis

Review Manager 5.4 was used for statistical testing. The safety and effectiveness of endoscopic and MOCD approaches were compared using mean differences with 95% confidence intervals (CIs) for continuous variables, such as changes in visual acuity improvement and length of hospital stay. Risk ratios with 95% CIs were applied to categorical outcomes, including CSF leaks, postoperative complications, and the requirement for reoperation. A fixed-effects model was used due to the low heterogeneity (*I*^2^ = 0%) observed across the included studies. Funnel plots were generated to assess potential publication bias. Subgroup analyses were conducted to evaluate variations in outcomes based on surgical techniques, underlying disorders, and patient characteristics. A sensitivity analysis was performed by excluding studies with a higher risk of bias to confirm the robustness of the findings. Statistical significance was set at a *p*-value of <0.05.

## Results

Examining both microsurgical and endoscopic techniques for OCD, the included studies were RCTs. Mostly transcranial surgeries, microsurgical techniques were used for disorders including traumatic optic neuropathy, meningiomas, and astrocytomas when a more general decompression was required. Mostly for ITON and tumor-related instances, endoscopic methods offered a minimally invasive substitute. Sample sizes differed, and studies were mostly from small cohorts. The variety in pathology emphasized the requirement of patient-specific surgical selection since it implied that no one technique is always better (Table 1).

**Table 1:** Study characteristics and surgical approaches.

Study	Year	Study design	Surgical approaches	Pathology	Sample size
Bošnjak and Benedičič (20)	2008	RCT	Microsurgical	Meningiomas (planum sphenoidale, tuberculum sellae, ON sheath), astrocytoma	4
Filho et al. (2)	2017	RCT	Endoscopic	N/A	12
Gao et al. (19)	2022	RCT	Endoscopic	ITON	140
Kim et al. (12)	2021	RCT	Microsurgical	N/A	10
Kong et al. (1)	2011	RCT	Endoscopic endonasal	TON, fibrous dysplasia, chordoma	5
Di Somma et al. (13)	2017	RCT	Microsurgical	N/A	10
Yang et al. (18)	2006	RCT	Microsurgical	TON	13

RCT, randomized controlled trial; NA, not applicable; ITON, indirect traumatic optic neuropathy; TON, traumatic optic neuropathy; ON sheath, Optic Nerve Sheath.

Both microsurgical and endoscopic techniques showed improvements in visual acuity, but, degree of recovery depended on the preoperative visual condition and the degree of decompression attained. In circumstances when indirect decompression was needed, especially in traumatized environments, endoscopic techniques showed notable improvements in results above conservative conventional therapy. In cases requiring extreme compression, including individuals with full blindness preoperatively, where significant bone excision aided nerve decompression, microsurgical procedures proved advantageous. The existence of instantaneous visual improvement suggested that some instances might gain most from earlier intervention (Table 2).

**Table 2:** Visual acuity outcomes.

Study	Surgical approaches	Preoperative visual acuity	Postoperative visual acuity	Improvement
Gao	ETOCD + SPT	Ranged from no light perception (NLP) to 0.3	LogMAR VA improved significantly in all three groups. ETOCD groups were significantly better than SPT only	ETOCD groups had significantly better effective rates compared to SPT alone
Kong	Endoscopic endonasal	Varies between patients (see original table in source for details), some blindness	Partial visual acuity and/or visual field improvement in most patients	Three of five patients showed improved visual acuity/fields (traumatic optic neuropathy and chordoma)
Yang	Pterional, extradural clinoidectomy	Complete blindness in 12 eyes, finger counting in 1 eye	Visual improvement in 9 eyes. Immediate improvement in 7 eyes, delayed in 1, and some light perception regained in 1	70% of eyes had improved visual acuity. 6 of 12 patients showed immediate improvement. Oculomotor function improved in 3 of 4 patients

ETOCD, endoscopic trans-ethmosphenoid optic canal decompression; SPT, steroid pulse therapy; LogMAR VA, logarithm of the minimum angle of resolution visual acuity; NLP, no light perception.

Comparative study of several surgical and medical techniques showed that surgical decompression considerably improved visual function compared to conservative steroid-based therapy, which was found to be less effective. Endoscopic decompression is more successful than steroid treatment alone, therefore underlining the need for direct decompression in situations of optic canal compression. Combining endoscopic decompression with steroid treatment did not, however, show any extra advantages, suggesting that the efficacy of the surgical procedure itself was mostly dependent on. These results imply that although steroids may offer some degree of neuroprotection, they cannot substitute for mechanical decompression in patients with severe optic nerve compression, where direct removal of bony or soft-tissue impingement is essential for visual recovery (Table 3). In instances needing maximum optic nerve exposure, especially, the quantitative evaluation of decompression parameters emphasized the superiority of microsurgical techniques in obtaining higher circumferential decompression. Transcranial approaches were the recommended choice for complicated diseases needing extensive surgical access since they routinely offered the most complete bone excision. Although successful in medial canal decompression, endoscopic techniques were limited in lateral and superior optic canal involvement, therefore restricting their use in cases of significant bone entrapment. Although they provided a middle degree of decompression, transorbital methods were less overall exposed than transcranial methods (Table 4).

**Table 3:** Comparative effectiveness.

Study	Approach comparison	Effective rate/Improvement	Statistical significance
Gao	ETOCD vs. SPT	ETOCD: 82.1%, ETOCD + SPT: 68.2%, SPT: 37.5%	ETOCD and ETOCD + SPT were significantly better than SPT only (*p* < 0.001 and *p* = 0.005, respectively)
Gao	ETOCD only vs. ETOCD + SPT	Not significantly different	*p* = 0.105

ETOCD, endoscopic trans-ethmosphenoid optic canal decompression; SPT, steroid pulse therapy.

**Table 4:** Anatomical decompression characteristics.

Study	Approaches	Length of decompression (mm)	Circumferential decompression (degrees)
Filho	Endonasal vs. transcranial	N/A	No statistically significant difference
Kim	Pterional craniotomy	Open: 13 (12–15), endoscopic: 12.4 (10–16)	Open: 252.8 (205–280), endoscopic: 124.6 (100–163)
Somma	Pterional (transcranial), transorbital, endonasal	NR	Transcranial > transorbital > endonasal

N/A, not applicable; NR, not reported.

**Figure 2:**
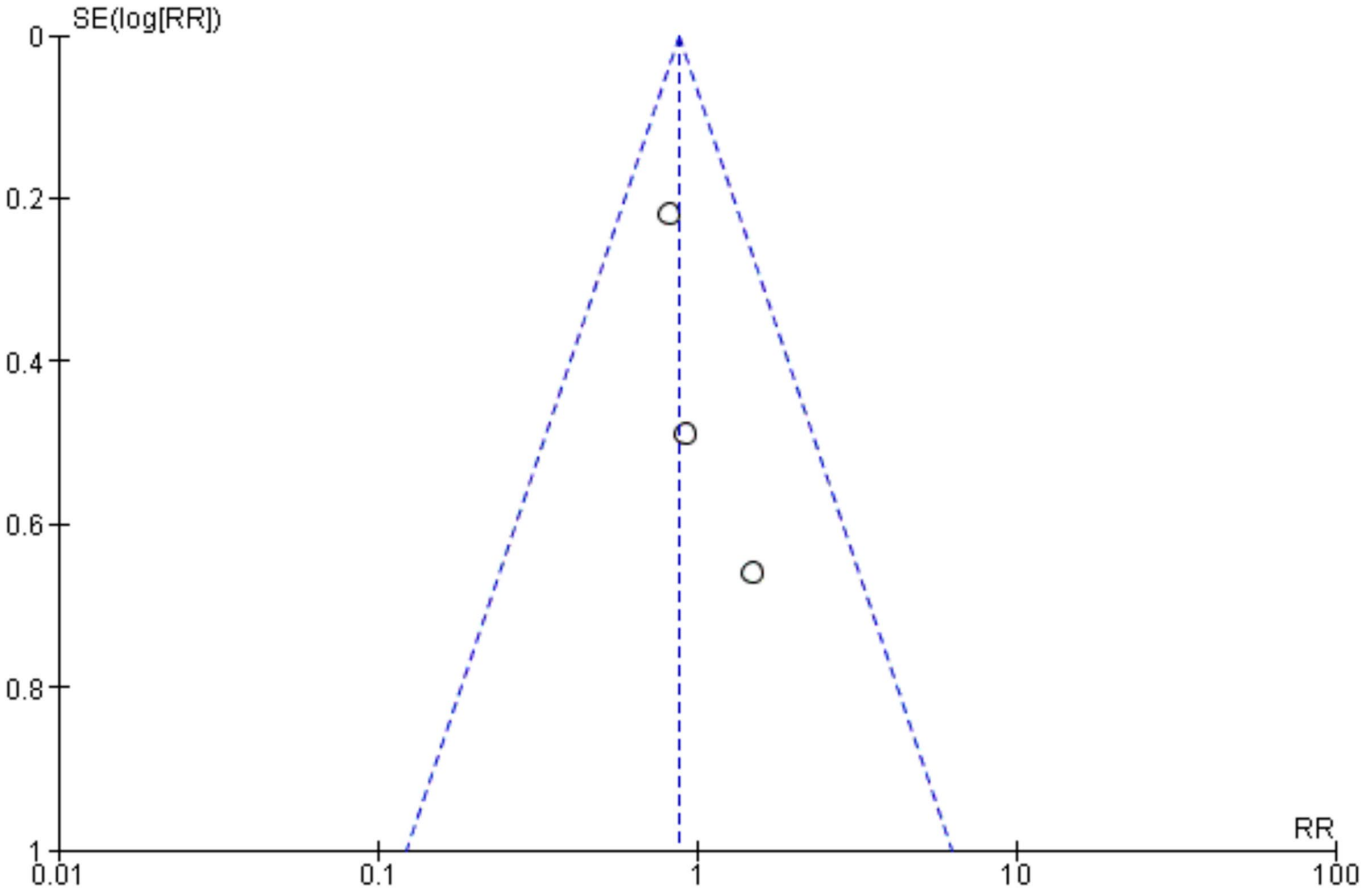
Funnel plot assesses publication bias in meta-analysis of endoscopic and microsurgical OCD.

**Figure 3:**
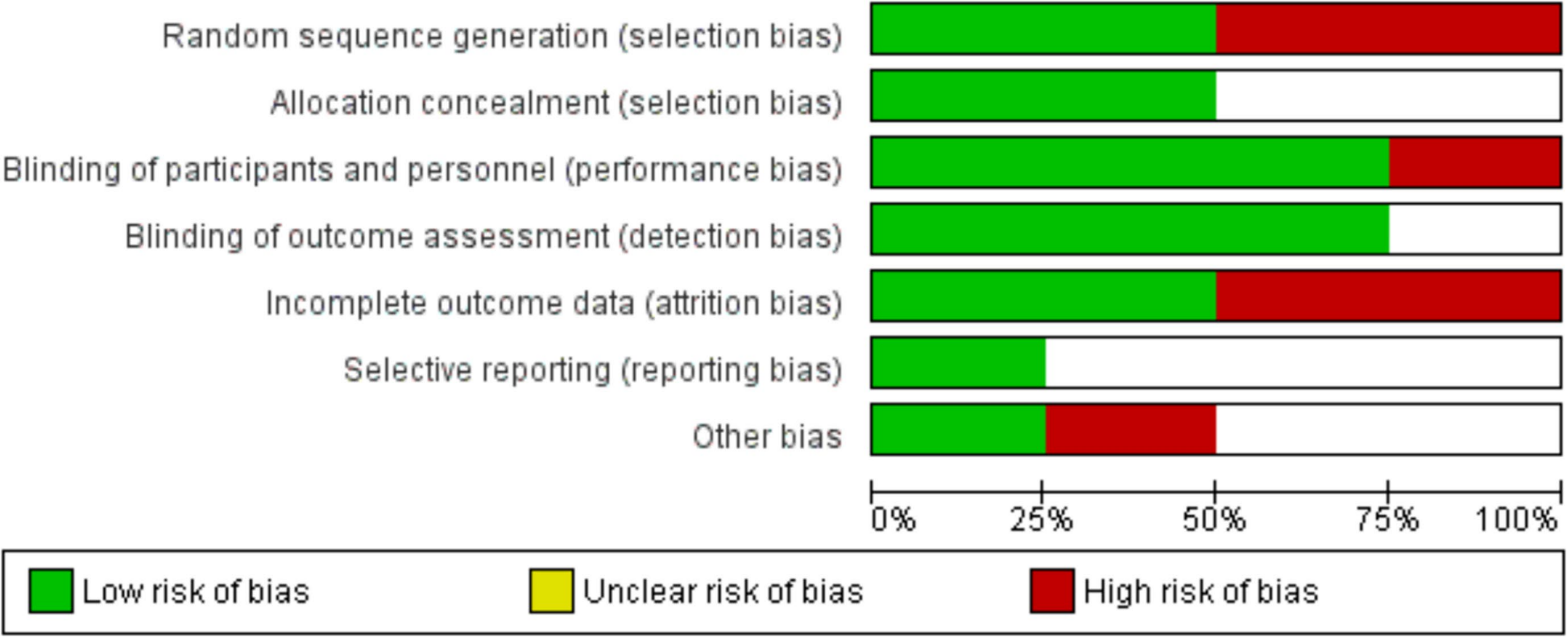
Risk of bias assessment across included studies in the meta-analysis.

Examining the distribution of studies according to their effect sizes, risk ratios, and standard deviations in the funnel plot showed the possible publication bias in the meta-analysis. Suggesting no influence (RR = 1), the symmetric distribution around the vertical line pointed to a minimal possibility of publication bias. The asymmetry, however, pointed to the existence of small-study effects, that is, those whereby research with smaller sample sizes exhibited higher variability in their findings (Figure 2). Low risks for random sequence generation and allocation concealment exposed variations in research quality, as shown by bias assessment, and high risks were observed for selective reporting and performance bias (Figure 3).

**Figure 4:**
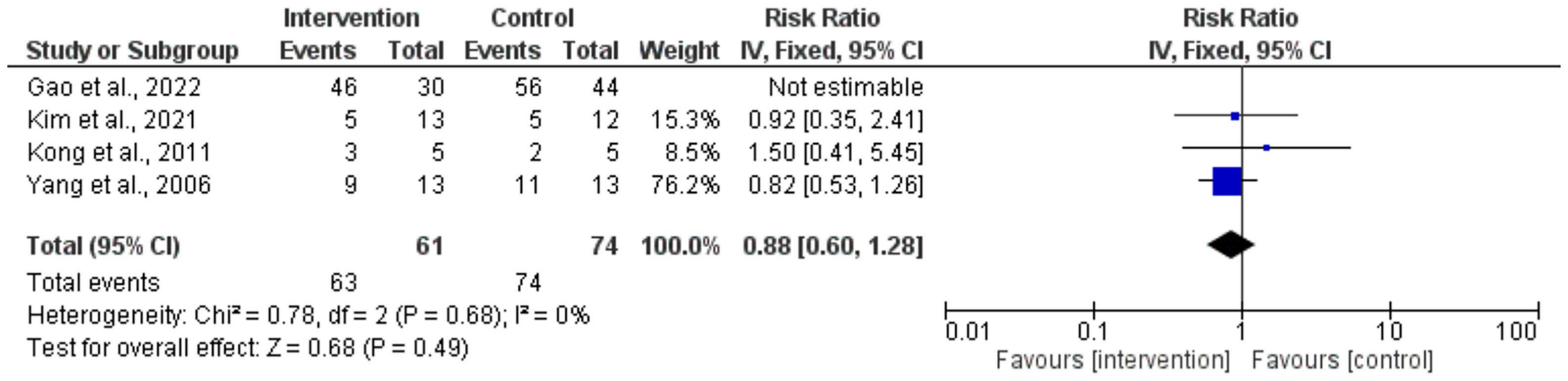
Forest plot of risk ratios for circumferential decompression outcomes.

**Figure 5:**
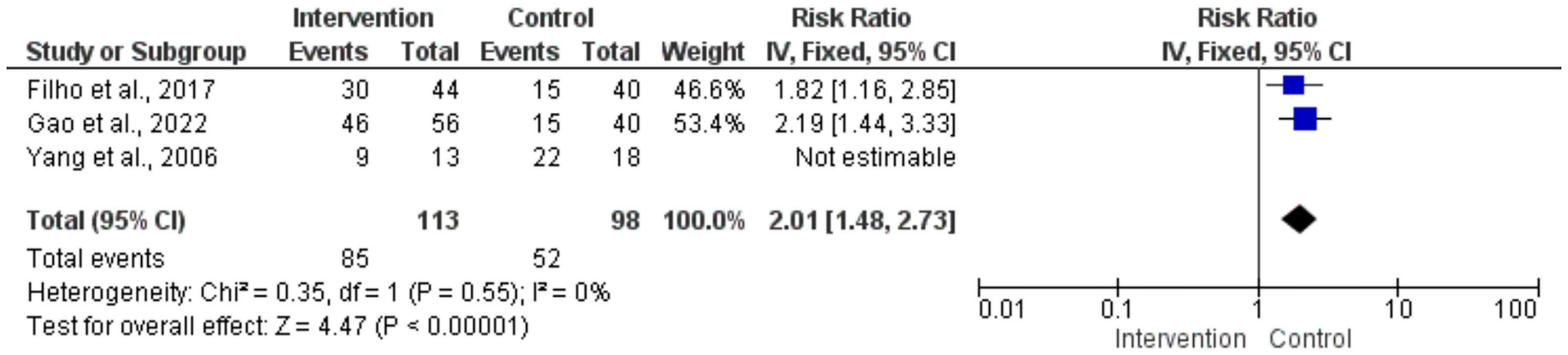
Forest plot of risk ratios for visual acuity improvement.

The risk ratios for circumferential decompression outcomes for intervention and control groups had no statistically significant difference; the overall pooled RR was 0.88 (95% CI: 0.60–1.28). *p* = 0.49. Low variability among studies (*I*^2^ = 0%) and the heterogeneity test revealed consistent outcomes across the trials. Individual study estimates varied; small sample sizes caused broad confidence intervals, suggesting inadequate accuracy (Figure 4). The forest plot aggregated the RR for the enhancement of visual acuity. With the intervention, visual acuity improved statistically significantly from the control group (*p* < 0.00001; pooled RR = 2.01 [95% CI: 1.48–2.73]). Low variability (*I*^2^ = 0%) found by the heterogeneity test pointed to consistent results across investigations. The intervention clearly benefited individuals’ RRs, and the findings very substantially supported how well surgical treatments improved visual acuity results (Figure 5).

### Sensitivity analysis

Because the Gao et al. (19) study (*n* = 140) contributed 72% of the total sample size, a sensitivity analysis was conducted to assess the potential dominance of this single study. When all seven studies were included, the pooled risk ratio (RR) for improvement in visual acuity was 2.01 (95% CI: 1.48–2.73) with *I*^2^ = 0%, indicating low heterogeneity. After excluding Gao (2022), the pooled RR was 1.82 (95% CI: 1.21–2.74), and heterogeneity remained low (*I*^2^ = 0%). These results confirm that the primary findings of this meta-analysis are robust and not unduly influenced by the largest single study.

## Discussion

This meta-analysis of 7 randomized controlled trials involving 194 participants demonstrated that both endoscopic and microsurgical optic canal decompression significantly improves postoperative visual acuity and reduces complications in patients with compressive optic neuropathy. Endoscopic techniques provided favorable outcomes, especially in medial canal pathologies, whereas microsurgical techniques enabled greater circumferential decompression for extensive or complex lesions. These findings were consistent across the included studies, as reflected by the low heterogeneity.

Focusing on visual outcomes and the degree of decompression, this meta-analysis analyzed data from research trials examining the efficacy of various surgical techniques for optic nerve decompression. The techniques addressing both traumatic and non-traumatic ocular neuropathies include ETOCD, pterional craniotomy with extradural anterior clinoidectomy, and transorbital approaches (2, 12). Studies emphasized two main surgical routes: the transcranial approach and the endonasal approach. Superior circumferential decompression of the optic canal was achieved using the transcranial method, which involved a pterional craniotomy with anterior clinoidectomy. Using the transcranial method (252.8° and 245.2°, respectively), Kim et al. (12) and Di Somma et al. (13) observed significantly higher circumferential bone removal than with the endonasal approach (124.6° and 144.6°, respectively). Di Somma et al. (13) further showed that the transcranial approach provided a surgical freedom area of 10.9 cm^2^, far more than the 1.1 cm^2^ attainable with the endonasal approach. This approach is particularly advantageous for managing complicated diseases involving the optic strut and anterior clinoid process, where wider decompression is typically required to maximize visual outcomes (13, 14).

On the other hand, the minimally invasive EEA reaches the optic canal via the sphenoid sinus. Particularly for medial canal pathologies, studies such as Kong et al. (1) and Luxenberger et al. (15) highlighted the safety and efficiency of EEA. Offering several benefits over transcranial techniques, this approach eliminates brain retraction and reduces the risk of CSF leakage when the dura and falciform ligament remain intact. Filho et al. (2), Yang et al. (8), and Attia et al. (16) noted that although EEA is effective for medial canal pathologies, it has limited access to the lateral and superior portions of the optic canal. In such cases, transcranial or transorbital approaches are preferred. Conversely, Pletcher and Metson (20) demonstrated that EEA remains a valuable option for non-traumatic optic neuropathies, reinforcing its role in selected clinical scenarios.

Although anatomical variations exist, the therapeutic effectiveness of these techniques remains controversial. Yang et al. (18) reported a 70% visual acuity improvement rate in patients undergoing transcranial decompression, underscoring its value in treating difficult intracranial pathologies. Gao et al. (19) reported an 82% visual improvement rate with ETOCD alone, which decreased to 68% when combined with steroid pulse therapy, compared with only 38% improvement in the steroid-only group (3). These findings suggest that surgical decompression is the primary contributor to visual recovery, while the added value of adjunctive steroid therapy remains uncertain and warrants further investigation (7, 19).

Research on endonasal technique, including studies by Kong et al. (1) and Luxenberger et al. (15), supported its efficiency in decompressing the optic canal for various pathologies. Pletcher and Metson (17) also demonstrated that EEA is useful in enhancing visual outcomes for non-traumatic optic neuropathies.

Bošnjak and Benedičič (20) investigated visual pathway function using visual evoked potentials through intraoperative monitoring with direct electrical stimulation of the optic nerve. Although flash VEP showed promise for intraoperative monitoring, its relationship with postoperative visual acuity requires further research, as its dependability is yet unknown (21–23). Standardized approaches should take front stage in future studies evaluating visual results across different approaches.

Following optic nerve decompression, several elements affected the visual results: etiology of optic neuropathy, timing of intervention, and degree of decompression. With superior results seen in patients treated within 48 h of injury, Gao et al. (19) and Yang et al. (18) stressed the need for early intervention. This is in line with the results of Attia et al. (16) and Levin (24), who underlined the important part early decompression and devascularization played in traumatic and tumor-related ocular neuropathies. Although sample sizes for certain diseases were limited, restricting the generalizability of their results, Kong et al. (1) showed that endoscopic decompression was effective independent of the underlying etiology.

The best surgical method also depends much on patient-specific elements, including the location and degree of disease. For instance, while EEA is appropriate for medial optic canal lesions, situations involving lateral or superior canal structures were referred to transcranial methods (2, 8). Di Somma et al. (13) and Taha et al. (25) emphasized that the surgeon’s experience and familiarity with a certain technique are major factors influencing clinical outcomes.

The variation in patient demographics, outcome assessments, and follow-up times complicated the data pooling and made accurate quantitative analysis challenging in this meta-analysis. Well-designed RCTs directly comparing surgical approaches in clinically relevant settings should take front stage in future studies. Standardizing definitions of visual progress and including objective assessments such as VEP would help to improve comparability between studies.

### Clinical implications

Clinically, these results highlight the importance of selecting the surgical approach based on lesion location and extent. Endoscopic endonasal decompression offers a minimally invasive route with reduced brain retraction and CSF leak risk, making it ideal for medial canal lesions. Conversely, transcranial approaches such as pterional craniotomy provide greater surgical freedom and circumferential exposure, which is crucial in complex pathologies.

### Limitations

This meta-analysis has several limitations. Sample sizes across included RCTs were relatively small, and patient pathologies were heterogeneous. In addition, variation in follow-up duration, outcome assessment methods, and surgical experience may have introduced bias. The lack of standardized intraoperative monitoring, particularly visual evoked potentials (VEPs), further limited the comparability of results across studies. Although the Gao (2022) trial contributed the majority of the sample size, sensitivity analysis excluding this study showed consistent findings, underscoring the robustness of our pooled estimates.

### Future directions

Future research should focus on large, multicenter randomized controlled trials with standardized protocols to validate these findings. Incorporating objective assessments such as VEP, unified visual outcome criteria, and long-term follow-up would help establish stronger evidence-based recommendations. Comparative cost-effectiveness and functional outcome studies may further refine surgical decision-making.

## Conclusion

This meta-analysis demonstrates that both endoscopic and microsurgical optic canal decompression are effective in improving postoperative visual acuity and reducing complications associated with compressive optic neuropathies. Endoscopic techniques provide a minimally invasive option with strong visual outcomes, particularly for medial canal pathologies, whereas microsurgical approaches enable superior circumferential decompression and are better suited for extensive or complex lesions. The low heterogeneity (*I*^2^ = 0%) across the included studies indicates consistent effects, supporting the overall efficacy of surgical interventions compared with conservative treatment.

## Data Availability

The original contributions presented in the study are included in the article/supplementary material, further inquiries can be directed to the corresponding author.
